# Core components of mental health stigma reduction interventions in low- and middle-income countries: a systematic review

**DOI:** 10.1017/S2045796020000797

**Published:** 2020-09-04

**Authors:** J. Clay, J. Eaton, P. C. Gronholm, M. Semrau, N. Votruba

**Affiliations:** 1 Centre for Global Mental Health and Centre for Implementation Science, Institute of Psychiatry, Psychology, and Neuroscience, King's College London, London, UK; 2 CBM Global and London School of Hygiene and Tropical Medicine, London, UK; 3 Centre for Global Health Research, Brighton and Sussex Medical School, Brighton, UK

**Keywords:** Discrimination, mental health, mental illness stigma, systematic reviews

## Abstract

**Aims:**

To identify and categorise core components of effective stigma reduction interventions in the field of mental health in low- and middle-income countries (LMICs) and compare these components across cultural contexts and between intervention characteristics.

**Methods:**

Seven databases were searched with a strategy including four categories of terms ('stigma’, ‘mental health’, ‘intervention’ and ‘low- and middle-income countries’). Additional methods included citation chaining of all papers identified for inclusion, consultation with experts and hand searching reference lists from other related reviews. Studies on interventions in LMICs aiming to reduce stigma related to mental health with a stigma-related outcome measure were included. All relevant intervention characteristics and components were extracted and a quality assessment was undertaken. A ‘best fit’ framework synthesis was used to organise data, followed by a narrative synthesis.

**Results:**

Fifty-six studies were included in this review, of which four were ineffective and analysed separately. A framework was developed which presents a new categorisation of stigma intervention components based on the included studies. Most interventions utilised multiple methods and of the 52 effective studies educational methods were used most frequently (*n* = 83), and both social contact (*n* = 8) and therapeutic methods (*n* = 3) were used infrequently. Most interventions (*n* = 42) based their intervention on medical knowledge, but a variety of other themes were addressed. All regions with LMICs were represented, but every region was dominated by studies from one country. Components varied between regions for most categories indicating variation between cultures, but only a minority of studies were developed in the local setting or culturally adapted.

**Conclusions:**

Our study suggests effective mental health stigma reduction interventions in LMICs have increased in quantity and quality over the past five years, and a wide variety of components have been utilised successfully – from creative methods to emphasis on recovery and strength of people with mental illness. Yet there is minimal mention of social contact, despite existing strong evidence for it. There is also a lack of robust research designs, a high number of short-term interventions and follow-up, nominal use of local expertise and the research is limited to a small number of LMICs. More research is needed to address these issues. Some congruity exists in components between cultures, but generally they vary widely. The review gives an in-depth overview of mental health stigma reduction core components, providing researchers in varied resource-poor settings additional knowledge to help with planning mental health stigma reduction interventions.

## Introduction

### Mental health stigma: a global problem

The term ‘stigma’ encompasses people's knowledge, negative attitudes and behaviours toward (or by) a certain group or individual deemed ‘unacceptably different’ (Scambler, 1998; Thornicroft *et al*., 2009). This paradigm links knowledge, attitude and behaviour, and has also been defined as problems in three domains: ignorance, prejudice and discrimination (Thornicroft *et al*., 2008).

Mental health stigma has been shown to be widespread globally, regardless of region (Pescosolido *et al*., 2013). For example, a 2009 study found rates of experienced discrimination by people with schizophrenia were high and consistent across 27 countries (Thornicroft *et al*., 2009).

The far-reaching negative impact of mental health-related stigma and discrimination has been extensively documented and has even been described by those with mental illness as ‘worse than the illness itself’ (Henderson and Thornicroft, 2009). There is evidence of negative impacts of stigma across multiple domains of life – for example, stigma is associated with reduced employment opportunities and corresponding poverty, relationship difficulties, reduced help-seeking behaviour and poorer quality health care (Corrigan, 2004; Jones *et al*., 2008; Thornicroft *et al*., 2009; Knaak *et al*., 2017). Additionally, people with mental illness often experience severe human rights abuses and lack of freedoms, which act as barriers to social inclusion (Patel *et al*., 2018).

Mental health stigma and discrimination have been identified as major factors for low levels of investment and political will for reform in many countries, which in turn reduces access to care and contributes to excess morbidity and mortality for people with mental illness (Saraceno *et al*., 2007). Given its wide-ranging detrimental impact, it is important to address stigma urgently and effectively (Henderson and Thornicroft, 2009).

### Stigma reduction interventions: state of the research

A substantial number of small-scale and short-term interventions have emerged in the past few decades focusing on reducing mental health stigma and several recent systematic reviews examine their effectiveness (Corrigan and Scott, 2012; Clement *et al*., 2013; Heim *et al*., 2018; Mehta *et al*., 2018; Mellor, 2018; Morgan *et al*., 2018).

Three overarching methods of reducing stigma have been conceptualised and tested in recent years: education (addressing myths and misconceptions), contact (direct or indirect interactions with people with the stigmatised condition) and protest (public demonstrations and campaigns against injustice) (Corrigan *et al*., 2001). The evidence indicates that there are a number of education and contact-based interventions which produce small to moderate effect sizes on stigma reduction, yet there is minimal evidence long-term and study quality is not always sufficient (Thornicroft *et al*., 2016; Gronholm *et al*., 2017; Morgan *et al*., 2018). The protest method has not shown evidence of effectiveness (Corrigan *et al*., 2001).

A few studies have been conducted to identify key ingredients for very specific contexts or stigma types (Pinfold *et al*., 2005; Mittal *et al*., 2012; Corrigan *et al*., 2013, 2014; Knaak *et al*., 2014). While these studies have provided useful setting-specific evidence, there have been no systematic reviews which identify core components of mental health stigma reduction interventions in LMICs or review their cultural variations. Similarly, there has been little research on how these components relate to other intervention factors – such as target population or type of stigma. A more detailed analysis would be helpful to understand what makes stigma reduction interventions effective in various contexts.

Investment by donor organisations for mental health has been noticeably increasing in high-income countries (HICs) over the past few years; for example, in January 2019 the Wellcome Trust announced £200 million in funding (Wellcome Trust, 2019). It is crucial that mental health researchers capitalise on this influx of support. In order to do so, they need to have sufficient evidence for how to design stigma reduction interventions both effectively and appropriately.

The scarcity of research into stigma reduction interventions in low- and middle-income countries (LMICs) is consistent with the wider mental health research gap in resource-poor settings (Collins *et al*., 2011; Thornicroft *et al*., 2017; Alonso *et al*., 2018). For example, only four out of 62 studies included in a recent mental health stigma-related review were from LMICs (Morgan *et al*., 2018). Intervention transferability from HICs to LMICs is context-dependent and cannot be assumed; therefore, there still is a vast gap in knowledge surrounding what works in diverse cultural contexts and why (Mehta *et al*., 2018). Given this systematic review's wide scope, cultural differences will be examined between geographic regions as classified by the World Bank.

This systematic review aims to address the scarcity of stigma research in LMICs by identifying and categorising core components of effective mental health stigma reduction interventions in LMICs and comparing these components across cultures.

## Methods

The protocol for this systematic review was registered on PROSPERO (ID 136008).

### Inclusion and exclusion criteria

This review included studies which contain interventions aiming to reduce any type of stigma related to mental health; this includes social/public stigma, self-stigma, anticipated, perceived, experienced stigma, or discrimination (see Table 1). Studies focusing on any other stigmatised condition were excluded, including HIV, neurological conditions, substance misuse and epilepsy. Interventions of all sizes, durations and effect sizes were included. There were no restrictions in terms of study participants. Inactive controls, treatment as usual controls and baseline assessments of intervention groups were all included, as long as outcome measures were taken before and after the intervention.

**Table 1.** Definitions of types of stigma and discrimination

Types of stigma	Definition
Social/public stigma	The prejudice and negative attitudes held by members of the public and/or society (Corrigan and Bink, 2005)
Self-stigma	Internalisation of prejudice and discrimination from social/public stigma (Corrigan and Bink, 2005)
Anticipated stigma	Expectations of bias from others (Stangl *et al*., 2019)
Perceived stigma	Perceptions of how the stigmatised group is treated by others (Stangl *et al*., 2019)
Experienced stigma	Experiences of being stigmatised by others (Stangl *et al*., 2019)
Discrimination	‘The behavioural result of prejudice’ (Corrigan and Bink, 2005)

All experimental designs were included, as long as they measured the effectiveness of stigma reduction interventions. To be included, interventions had to have been conducted in countries classified as LMIC by the World Bank (The World Bank, 2019). There were no publishing date restrictions. Following established frameworks on conceptualising stigma, studies had to include at least one measure of mental health-related stigma linked to knowledge, attitudes or behaviour (Corrigan and Scott, 2012; Thornicroft *et al*., 2016).

### Search strategy

The database search strategy was developed using earlier stigma-related systematic reviews as a guide (Heim *et al*., 2018; Mehta *et al*., 2018; Morgan *et al*., 2018; Kemp *et al*., 2019) and used both subject headings and keywords. Four categories of terms ('stigma’, ‘mental health’, ‘intervention’, ‘low- and middle-income countries’) were expanded with synonyms and related subject headings, connected within categories with ‘OR’ and between categories with ‘AND’. The full search strategy for MEDLINE, exemplifying this process, is provided within online Supplementary Material. Searches were restricted to English and Spanish, and to humans.

The following seven databases were searched on 13 May 2019: Cochrane Central Register of Controlled Trials (CENTRAL), MEDLINE, Cumulative Index to Nursing and Allied Health Literature (CINAHL), Global Health, PsycINFO, EMBASE and Scopus.

Additional search methods comprised of citation checking, hand checking reference lists from other related systematic reviews on stigma, and experts in the field (NV, JE) were consulted to identify any missing papers or grey literature.

### Study selection

All titles and abstracts were screened against the inclusion criteria by the lead author (JC), and 10% of titles and abstracts were screened by a second reviewer to establish consistency. Disagreements were resolved by discussion. The study supervisor with knowledge of the review topic (NV) decided any on unresolved discrepancies.

Full-text versions of papers were retrieved for all potentially relevant studies and screened against the inclusion criteria. If the full text of a study was not available, the author was contacted. If there was no reply, the study was excluded. A third reviewer screened 10% of full-text papers.

### Quality assessment

Assessment of quality and risk of bias across studies was conducted with the Mixed Methods Appraisal Tool (MMAT) (Hong *et al.*, 2018). This tool was chosen because multiple study designs were included in this review, and the MMAT has five unique criteria for each design in addition to two core criteria.

All studies marked for inclusion were assessed for quality and the third reviewer separately assessed 10% for consistency. For a study to be included, the two standardised core criteria had to be met. As recommended by the MMAT, studies of poor or very poor quality – two or less of five criteria met – were included in the main analysis but later separated out.

### Data extraction

The framework for data extraction was developed a priori. Data was extracted on general study information (e.g. author, year), study characteristics (e.g. design, aims), study methods (e.g. methods of recruitment), intervention characteristics (e.g. intervention methods, dissemination medium), results (e.g. outcomes) and methodological quality. A full list of fields is provided in online Supplementary Material. Missing data was requested from authors where possible.

### Data analysis

Only interventions which produced at least a partially positive effect for stigma-related outcomes were included in the main analysis. Ineffective interventions were described separately, to demonstrate how their components differed.

With previously conducted research and frameworks as a guide, a ‘best fit’ framework synthesis was chosen as the main method of analysis for this review (Carroll *et al*., 2013). As the most broadly encompassing framework, Corrigan's five categories of stigma reduction ingredients were the starting point for the synthesis: programme design, targeting, staffing, messaging and follow-up/evaluation. (Corrigan *et al*., 2013).

In order to address the cultural focus of this review, the sixth category of components was added: Culture. Data extraction fields related to this included: detail on the intervention rationale, theory or origin (where the content came from), as well as whether the publications mention any kind of cultural adaptation or taking account of local beliefs. This data helped determine the influence of culture on the intervention and provide detail on transferability. World Bank regions were also analysed individually, in order to describe differences in components by region.

Data was extracted from each study based on the authors' literal descriptions in the publications, without a priori labels. A codebook was then created for each data extraction worksheet field, grouping information within the categories using inductive thematic analysis grounded in the extracted data. The framework synthesis then allowed for the expansion of Corrigan's categories (Carroll *et al*., 2011) and the creation of a new framework (see Fig. 2). A narrative synthesis was used to explain the coded data. The final overview of components therefore only included those which the publication reported on.

## Results

### Search results

**Fig. 1.**
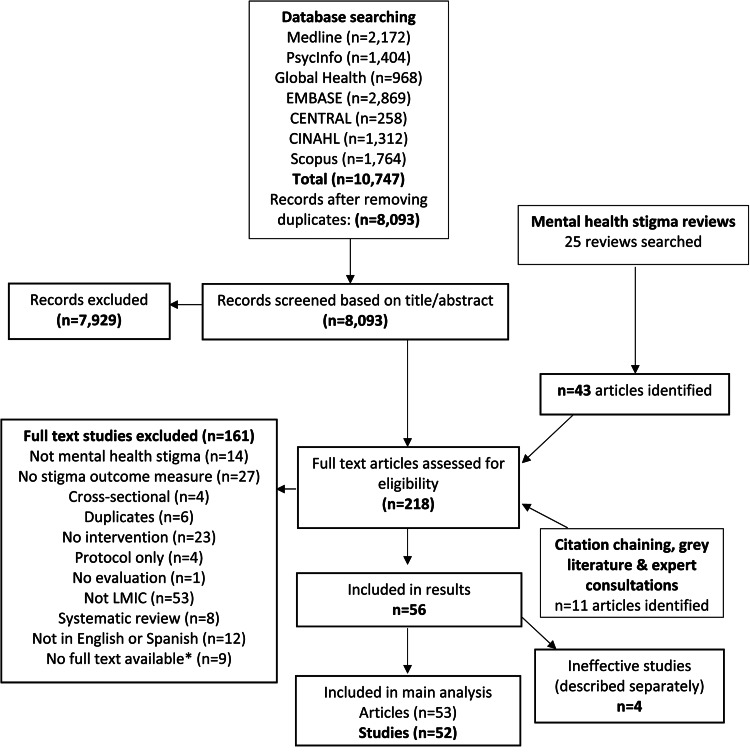
PRISMA flowchart of selection of articles and sources included in the review. *Authors contacted with no response

The final search produced 56 studies (57 articles) which were deemed eligible for inclusion (see Fig. 1). Four studies were ineffective and analysed separately, to demonstrate how their components differed.

### Study characteristics

See Table 2 for key characteristics of each study.

**Table 2.** Key characteristics and components of included studies

Author (year)^a^	Study design	Country	Target pop^b^	Target condition (type)^c^	*n*^d^	% complete outcome data	Duration	Overall quality^e^	Intervention methods^f^	Content detail	Lived exp	Cultural adaptation	Follow-up time points^g^	Outcome measure(s) focal point(s) (validation)^h^	Overall effectiveness for stigma-related outcomes
Abayomi *et al*. (2013)	Pre/post	Nigeria	HCW	Mental health (SP)	60	51%	6 weeks	Moderate	L/P, Train	Medical	No	Translation only	End only	Attitudes(*)	Yes, significant effect
Adelekan *et al*. (2001) (–)	Pre/post	Nigeria	HCW	Mental health (SP)	43	62%	No info	Poor	L/P, Train	Medical, Prevention	No	No	End only	Attitudes	No evidence of effectiveness
Ahuja *et al*. (2017)	Pre/post	India	Students	Mental health (SP)	50	100%	1 session	Moderate	L/P, T/D, Disc	Myths, Celebs, Strength	Yes	Yes	End; 1 week after	Attitudes(*)	Yes, significant effect
Altindag *et al*. (2006)	Non-randomised trial	Turkey	Students	Schizophrenia (SP)	60	77%	1 day	High	L/P, Disc, DSC, Film	Medical, Myths, Strength	Yes	No	End; 1 month after	Attitudes(*)	Yes, significant effect
Amaresha *et al*. (2018)	Non-randomised trial	India	F/C	Schizophrenia (SE)	80	78%	Unclear	High	L/P, Disc	Medical, Emotional	Yes	Yes	End; 1 month; 3 months after	Knowledge(*); Self-stigma(*)	Yes, significant effect
Amna *et al*. (2016)	Non-randomised trial	Indonesia	Students	PTSD (SP)	121	Unknown	No info	Poor	L/P, SS, Disc	Medical, Facts	No	No	End only	Attitudes(*)	Yes, significant effect
Armstrong *et al*. (2011)	Pre/post	India	HCW	Mental health (SP)	70	94.30%	4 days	High	L/P, Train, Role	Facts, Medical, MHFA, Stigma	No	No	End; 3 months after	Knowledge & Attitudes(*)	Very small positive effect; mixed results
Ayano *et al*. (2017)	Pre/post	Ethiopia	HCW	Mental health (SP)	94	100%	5 days	Moderate	L/P, Vid	Medical	No	No	End only	Knowledge & Attitudes(*)	Yes, significant effect
Ayonrinde *et al*. (1976) (–)	Pre/post	Nigeria	HCW	Mental health (SP)	72	Unknown	No info	Very poor	L/P, Disc	Medical	No	No	End only	Knowledge & Attitudes(*)	No evidence of effectiveness; had a negative effect
Bella-Awusah *et al*. (2014)	Non-randomised trial	Nigeria	Students	Mental health (SP)	154	94%	1 session	High	L/P, Disc,	Facts, Medical, Stigma, Media	No	Yes	End; 6 months after	Knowledge & Attitudes(*)	Significant effect for knowledge, but not attitudes/social distance
Berlim *et al*. (2007)	Pre/post	Brazil	HCW	Suicide (SP)	142	100%	1 session	Moderate	L/P, Train, Disc	Facts, Medical	No	No	End only	Attitudes(*)	Yes, significant effect
Bosoni *et al*. (2017)	Pre/post	Brazil	Public	Schizophrenia (SP)	48	57%	1 session	Poor	Vid	Facts, Medical, Stigma, Recovery	No	Yes	End; 1 month; 3 months after	Stereotyped views(*)	Yes, significant effect
Botega *et al*. (2007)	Pre/post	Brazil	HCW	Suicide (SP)	317	80%	2 sessions (unclear)	Moderate	L/P, Disc, RA, Train	Stigma, Facts, Medical	No	Translation only	End; 3 months; 6 months after	Attitudes(*)	Yes, significant effect for 2/3 subscales
Chinnayya *et al*. (1990)	Pre/post	India	HCW	Mental health (SP)	150	100%	1 week	High	L/P, Case, Role	No content detail	No	Translation only	End only	Attitudes(*)	Yes, significant effect
Cuhadar *et al*. (2014)	Individual RCT	Turkey	SU	Bipolar (SE)	63	74%	7 weeks	Moderate	L/P, Disc, Share	Medical, Facts, Stigma	Yes	No	End only	Self-stigma(*)	Yes, significant effect
da Silva *et al*. (2011)	Pre/post	Brazil	HCW	Suicide (SP)	230	58%	Unclear	Poor	L/P, Disc, Case, Share	Medical, Facts, Stigma, Prevention	No	Minimal	End only	Attitudes(*); Knowledge	Yes, significant effect
Demiroren *et al*. (2016) (–)	Non-randomised trial	Turkey	Students	Mental health (SP)	190	95%	2 sessions (unclear)	High	Reflect	No additional content	No	Yes	End only	Attitudes(*)	No evidence of effectiveness
Dharitri *et al*. (2015)	Pre/post	India	Public, F/C	Mental health (SP)	1112	94%	Unclear	High	L/P, Poster, Q&A, Print, T/D	Medical, Myths, Stigma	No	Yes	In 1st month of intervention; 3 months after	Attitudes(*)	Yes, significant effect
Domínguez-Martinez *et al*. (2017)	Pre/post	Mexico	F/C	Mental health (SP)	291	79%	12 weeks	Moderate	L/P, Disc	Recovery, Medical, Emotional, Strength	Yes	Minimal	End only	Knowledge(*)	Yes, significant effect
Duman *et al*. (2017)	Non-randomised trial	Turkey	Students	Mental health (SP)	256	78%	2-3 months	Poor	L/P, Disc, Film, Obs, DSC	Stigma	Yes	Yes	End only	Attitudes(*)	Yes, significant effect
El-Nahas *et al*. (2018)	Individual RCT	Egypt	F/C	Schizophrenia (SP)	60	83%	6 months	High	L/P, Disc, Train	Medical, Emotional	No	Yes	End only	Attitudes(*); Knowledge(*)	Yes, significant effect
Fernandez *et al*. (2016)	Individual RCT	Malaysia	Students	Mental health (SP)	102	100%	1 day	High	L/P, DSC, VSC	Medical, Facts, Stigma, Myths, Recovery	Yes	No	End; 1 month after	Attitudes & Behaviour(*)	Yes, significant effect
Finkelstein *et al*. (2008)	Individual RCT	Russia	Students	Mental health (SP)	193	79%	1 session	Moderate	L/P, Quiz, SSC	Myths, Medical, Facts, Recovery, Strength	No	No	End; 6 months after	Attitudes(*); Attitudes(*); Knowledge(*)	Yes, significant effect
Goyal *et al*. (2013)	Pre/post	India	Students	Depression (SP)	100	95%	1 session	Moderate	Print	Facts, Medical, Myths	No	Yes	1 week after baseline only	Knowledge & Attitudes(*)	Significant effect for knowledge, but not attitudes
Hofmann-Broussard *et al*. (2017)	Pre/post	India	HCW	Mental health (SP)	56	100%	4 days	High	L/P, Disc, DSC	Facts, Medical, Recovery	Yes	No	End only	Knowledge(*); Attitudes(*)	Significant effect for psychosis, but not depression
Iheanacho *et al*. (2014)	Pre/post	Nigeria	Students	Mental health (SP)	83	69%	4 days	Poor	L/P, Disc, Role	Medical, Facts	No	No	End only	Attitudes(*)	Yes, significant effect
Keynejad *et al*. (2016)	Mixed methods pre/post	Somaliland (SL)	Students	Mental health (SP)	20 SL (24 UK)	39%	20 weeks	High	Web, Forum, L/P, IM	Medical, Culture, Facts	No	Yes	End only	Attitudes(*); Attitudes(*)	Yes, for SL students (not UK students)
Kutcher *et al*. (2015)	Pre/post	Malawi	Teachers	Mental health (SP)	218	88%	3 days	Moderate	Train, Disc	Facts, Medical, Stigma	No	Yes	End only	Knowledge(*); Attitudes(*)	Yes, significant effect
Kutcher *et al*. (2016)	Pre/post	Tanzania	Teachers	Mental health (SP)	61	62%	3 days	Very poor	L/P, SS, Disc	Stigma, Medical, Facts, Recovery	No	Yes	End only	Knowledge(*); Attitudes(*); Knowledge(*)	Significant effect for knowledge and attitudes, but not comfort levels
Li *et al*. (2014)	Pre/post	China	HCW	Mental health (SP)	99	90%	1 day	Moderate	L/P, Train, Share	Medical, Stigma	No	No	End only	Knowledge; Behaviour(*); Attitudes(*)	Significant effect for discrimination and attitudes, but not for knowledge
Li *et al*. (2018)	Cluster RCT	China	SU	Schizophrenia (SE)	384	84%	9 months	Moderate	L/P, Train, Disc, CBT	Medical, Facts, Emotional, Stigma	Yes	Yes	6 months into intervention; at end	Internalised stigma(*); experienced stigma(*)	Significant effect for anticipated discrimination and overcoming stigma, but not self-stigma
Li *et al*. (2019)	Cluster RCT	China	HCW	Mental health (SP)	384	76%	1 session	Poor	L/P, Train	Stigma, Recovery	No	No	End only	Perceived discrimination(*); Attitudes(*); Knowledge(*)	Significant effect for perceived discrimination and attitudes, but not knowledge
Makanjuola *et al*. (2012)	Pre/post	Nigeria	Teachers	Mental health (SP)	24	Unknown	5 days	Very poor	L/P, Train, Disc, Role, Vid	Medical, Facts, Emotional, Policy	No	No	End only	Knowledge & Attitudes	Yes, significant effect
Maulik *et al*. (2017, 2019)	Mixed methods pre/post	India	Public	Mental health (SP)	1576	73%	3 months	Moderate	Public, Print, DiscPub, Disc, VSC, Vid, T/D	Medical, Stigma	Yes	Yes	End; 2 years after baseline	Knowledge & Attitudes & Behaviour(*)	End: Significant effect for attitudes and behaviour, but not knowledge 2 years: Yes, significant effect
Mutiso *et al*. (2019)	Pre/post	Kenya	HCW, SU	Mental health (SE)	2305	59%	5 days	High	L/P, Train, Case, Disc, Role	Medical, Stigma	Yes	Yes	6 months after	Experienced stigma(*)	Yes, significant effect
Ng *et al*. (2017)	Pre/post	Malaysia	HCW	Mental health (SP)	206	99%	1 session	High	Vid	Stigma, Myths, Celebs, Strength, Recovery, Facts	Yes	Translation only	End only	Attitudes & Behaviour(*)	Yes, significant effect
Ngoc *et al*. (2016)	Individual RCT	Vietnam	SU	Schizophrenia (SE)	59	81%	1-2 weeks	Moderate	L/P, Disc	Medical, Stigma, Recovery, Emotional	Yes	Yes	6 months after enrolment only	Attitudes(*)	Yes, significant effect
Oduguwa *et al*. (2017)	Individual RCT	Nigeria	Students	Mental health (SP)	205	66%	3 days	Moderate	L/P, Disc, Role	Facts, Medical, Stigma	No	No	End; 3 weeks after	Knowledge & Attitudes(*)	Significant effect for knowledge, but not for attitudes or social distance
Pejovic-Milovancevic *et al*. (2009)	Pre/post	Serbia	Students	CAMH (SP)	63	100%	6 weeks	Poor	L/P, Disc, WS	Facts, Medical, Stigma, Myths	No	No	6 months after	Attitudes	Yes, significant effect
Pereira *et al*. (2015)	Cluster RCT	Brazil	Teachers	CAMH (SP)	176	65%	3 weeks	Moderate	Web, L/P, Vid, Print, WC	Medical, Myths, Stigma	No	Yes	End only	Knowledge & Attitudes	Significant effect for knowledge and stigmatised concepts, but not attitudes
Rahayuni *et al*. (2013)	Non-randomised trial	Indonesia	F/C	Schizophrenia (SP)	38	97%	2.5 weeks	High	L/P, BS, Case, Role, Disc, Print, Vid	No content detail	No	Yes	End only	Attitudes(*)	Yes, significant effect
Rahman *et al*. (1998)	Cluster RCT	Pakistan	Students, Public	Mental health (SP)	100	100%	6 months	High	L/P, Disc, T/D	Facts	No	Yes	4 months after baseline only	Knowledge & Attitudes(*)	Yes, significant effect
Ran *et al*. (2003)	Cluster RCT	China	F/C	Schizophrenia (SP)	357	91%	9 months	High	L/P, Disc, WS, Share, Train	Facts, Emotional	Yes	Yes	End only	Attitudes(*)	Yes, significant effect
Ravindran *et al*. (2018)	Non-randomised trial	Nicaragua	Students	Mental health (SP)	913	67%	12 weeks	High	Web, Vid, Share, L/P, Disc	Stigma, Facts, Medical	No	Yes	End only	Knowledge & Attitudes(*)	Yes, significant effect
Rong *et al*. (2011)	Cluster RCT	China	Students	Depression (SP)	205	Unknown	10 days	Moderate	L/P, Disc, BS, DiscPub, Art, Vid	Medical, Facts, Policy, Celebs, Strength	Yes	Yes	2 weeks; 1 month; 6 months (after baseline)	Knowledge & Attitudes(*); Attitudes(*)	Yes, significant effect
Sadik *et al*. (2011)	Pre/post	Iraq	HCW	Mental health (SP)	317	100%	2 weeks	High	L/P, Disc, Train, Role, Vid, Visits, CD, Print	Medical, Policy	Yes	Yes	End only	Knowledge & Attitudes & Behaviour(*)	Yes, significant effect
Sanhori *et al*. (2019) (-)	Pre/post	Sudan	IDP	Mental health (SP)	1549	82%	1 session	High	L/P, Q&A, Disc, T/D	Facts, Medical, Recovery, Stigma	No	No	1 year after baseline only	Attitudes(*)	No evidence of effectiveness
Shah *et al*. (2015)	Pre/post	India	HCW	Mental health (SP)	150	Unknown	2 years	Poor	Train, Disc, Role, DSC, Obs, Quiz	Facts, Medical	Yes	No	End only	Knowledge & Attitudes	Yes, significant effect
Shamsaei *et al*. (2018)	Pre/post	Iran	F/C	Mental health (SP)	43	100%	1 day	Poor	L/P, Print, Share, Disc	Stigma, Medical	No	No	1 week after	Attitudes(*)	Yes, significant effect
Sibeko *et al*. (2018)	Mixed methods pre/post	South Africa	HCW	Mental health (SP)	58	69%	8 sessions (unclear)	High	L/P, Disc, Reflect	Culture, Medical	No	Yes	End; 3 months after	Knowledge(*); Attitudes(*)	Yes, significant effect
Tong *et al*. (2019)	Individual RCT	China	SU	Depression (SE)	90	97%	8 weeks	High	GCBT	Facts, Emotional, Stigma	Yes	No	End only	Stigma- no further detail(*)	Yes, significant effect
Ucok *et al*. (2006)	Pre/post	Turkey	HCW	Schizophrenia (SP)	106	50%	1 session	Very poor	L/P, Disc, Print	Medical, Stigma	No	No	3 months after	Attitudes	Significant effect for 5/16 items; mixed results
Vaghee *et al*. (2015)	Cluster RCT	Iran	F/C	Schizophrenia (SE)	90	93%	4 days	High	Film, Disc, L/P, Q&A	Emotional, Recovery, Facts	Yes	No	1 month after	Internalised stigma(*)	Yes, significant effect
Worakul *et al*. (2007)	Pre/post	Thailand	F/C	Schizophrenia (SP)	91	100%	1 day	Poor	L/P, Disc, Film	Medical, Emotional	No	Yes	End only	Knowledge; Attitudes	Yes, significant effect
Yan *et al*. (2018)	Individual RCT	China	Students	Anorexia nervosa (SP)	76	Unknown	2 sessions (unclear)	Poor	Case, Web	In/Out-group	No	Yes	End only	Attitudes(*)	Yes, significant effect for in-group
Yılmaz *et al*. (2018)	Non-randomised trial	Turkey	SU	Schizophrenia (SE)	80	86%	6 weeks	High	Train, L/P, Role, Share	Emotional, Facts, Medical, Stigma	Yes	No	End only	Internalised stigma(*)	Yes, significant effect

Note: Studies with a ‘(–)’ in the first column after the author's name were ineffective for stigma-related outcomes and excluded from the main analysis.

a Year, year of publication.

b Target population: HCW, health care worker; F/C, family/caregiver; Public, the general public; SU, service user; IDP, internally displaced persons.

c Target condition (type): SP, stigma practices (expressed by those perpetuating stigma); SE, Stigma experiences (those felt by the stigmatised individual); CAMH, child and adolescent mental health; PTSD, post-traumatic stress disorder.

d *N,* number of participants at baseline.

e Study quality was based on the Mixed Methods Appraisal Tool (MMAT).

f Intervention methods: see Table 3 for component codes.

g Follow-up time points: after baseline. End = immediately after intervention; after = after the end of the intervention.

h Outcome measure focal points = what are study outcome measures looking at as a proxy for stigma. Validation: (*) = measure is validated in some way; no ‘(*)’ = measure was developed ad hoc by authors with no validation OR no info given. One measure which addresses more than one focal point connects with ‘&’; two separate measures are indicated with ‘;’. Full names/descriptions of measures available upon request.

The quality of studies varied, with 38 studies (73%) fulfilling at least three of five MMAT quality criteria (considered moderate and high-quality studies). Eleven studies (21%) were of poor quality with one to three of criteria fulfilled, and three (6%) were of very poor quality (i.e. no criteria fulfilled).

The majority of studies were conducted in East Asia and Pacific (*n* = 13, 25%) or Sub-Saharan Africa (*n* = 11, 21%), but all World Bank regions were represented apart from North America, due to the review's LMIC restriction. Studies took place in 24 countries overall. Only two studies were published prior to 2000, and about half (*n* = 26) have been published since 2016; 12 were published in 2018 or 2019. Almost a third of studies (*n* = 15, 29%) were individual or cluster randomised controlled trials (RCTs) and 11 of these (73%) were published since 2014. The majority of studies (*n* = 27, 52%) were pre/post studies.

Most studies (*n* = 29, 56%) targeted general mental health stigma. Twelve studies looked at schizophrenia, three studies focused on suicide, three on depression, two on child and adolescent mental health, and one each on bipolar disorder, anorexia nervosa and post-traumatic stress disorder.

The majority of studies (*n* = 30, 58%) had their final outcome assessment directly at the end of the intervention, but 14 of these had an intervention duration of 4 weeks or longer. Fifteen (29%) followed up after 2 months or longer. Thirty-nine articles (74%) found a significant positive result for all main stigma outcomes and 13 (25%) found at least a small positive result for some but not all stigma outcomes. Of these 13, ten lasted less than a week.

Among the moderate or high-quality studies (*n* = 38), 14 (37%) followed up at least 1 month after the intervention finished and ten of these (71%) still found a significant positive effect on stigma outcomes at the later final assessment, indicating that it is possible to maintain stigma reduction over the medium term.

### Intervention components and categorisation

**Fig. 2.**
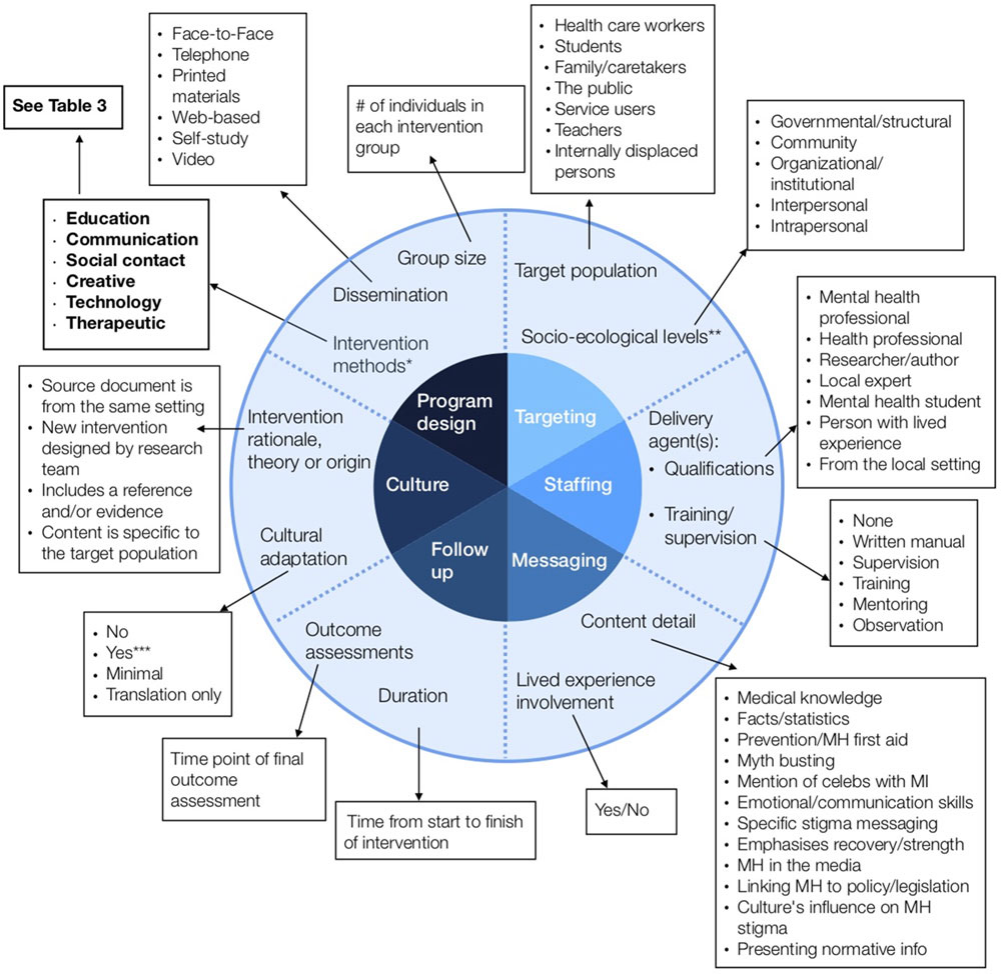
Framework of core components of anti-stigma interventions in low- and middle- income countries. The inner circle represents six overarching ‘categories’; the outer circle represents ‘components’ within each category; the boxes represent ‘sub-components’ within each component. Intervention methods are further broken down into ‘elements’ in Table 3. This framework of core components of anti-stigma interventions was developed by the authors as a composite of other analysis frames (Heijnders & Van Der Meij, 2006; Corrigan *et al*., 2013). *Intervention methods: the six sub-components are further coded into 32 elements; see Table 3. **Socio-ecological levels: based on Heijnders' framework (Heijnders & Van Der Meij, 2006). ***Cultural adaptation: ‘yes’ = local beliefs/culture are taken into account; the intervention at least partially originated from the local context; or, the intervention was piloted/field-tested

From the best-fit framework synthesis, the categorisation of components was developed into a new framework (see Fig. 2) which was used to organise and analyse extracted data. This produced six categories of components: programme design, targeting, staffing, messaging, follow-up and culture, described in further detail below. Full data is available upon request.

#### Programme design

The programme design category captured group size, dissemination and intervention method components. As the quantity and variety of intervention method sub-components identified through thematic analysis was vast, these were further organised into ‘elements’ (see Table 3). Most studies (*n* = 49, 94%) utilised at least one educational component and only eight did not include the most common element, ‘lectures/presentations’. Almost all (*n* = 48, 92%) reported using more than one element and 20 studies (38%) used four or more intervention method elements.

**Table 3.** ‘Intervention methods’ sub-components and elements used in anti-stigma interventions in low-and middle-income countries

Intervention methods sub-components	Elements	Code
Education	Lectures/presentations	L/P
	Training	Train
	Case studies	Case
	Printed material	Print
	Quiz	Quiz
	Self-study	SS
	Public campaign	Public
	Visits to mental health units	Visits
	Observation	Obs
Communication	Discussion	Disc
	Reflection	Reflect
	Sharing of experiences	Share
	Posters	Poster
	Q&A	Q&A
	Workshop	WS
	Discussions with the public	DiscPub
	Brainstorming strategies	BS
Social contact	Direct social contact	DSC
	Simulation of social contact	SSC
	Video of social contact	VSC
Creative	Film screening	Film
	Roleplay	Role
	Theatre/dance	T/D
	Art	Art
Technology	Videos	Vid
	CDs	CD
	Website	Web
	Web forum	Forum
	Web conference	WC
	Instant messaging	IM
Therapeutic	CBT	CBT
	Group CBT	GCBT

Across all studies, as shown in Table 3, educational sub-components were documented most frequently as a method (*n* = 83), followed by communication (*n* = 58), technology (*n* = 21), creative (*n* = 19), social contact (*n* = 8) and therapeutic (*n* = 3). As for group size, the majority were between 11 and 29 (*n* = 15, 29%) and between 30 and 99 (*n* = 8, 15%) or it was unclear/unreported (*n* = 21, 40%). Of the 39 interventions effective for all stigma outcomes, only one described using educational methods only, and just 14 (36%) used solely education and communication methods.

#### Targeting

The most frequent target populations were health care workers and students (both *n* = 16, 31%), although some studies focused on more than one group.

#### Staffing

The most common delivery agents were mental health professionals (MHP) (*n* = 23, 44%). Of these, only six explicitly mentioned that the MHP came from inside the setting where the research took place. Two (4%) defined at least one of their delivery agents as someone with lived experience. Of the total, 54% of studies (*n* = 28) did not mention any delivery agent training or supervision.

#### Messaging

The content of messaging did vary, but most interventions (*n* = 42, 81%) described their intervention as being based on medical knowledge, and only half of the studies (*n* = 26) explicitly mentioned stigma or discrimination in their description. While this does not necessarily indicate that a discussion of stigma was excluded from the other 26 interventions, it was not reported. A variety of other themes were addressed in smaller numbers including teaching emotional/communication skills (*n* = 11); myth-busting (*n* = 9); emphasising recovery and strength of people with mental illness (*n* = 13); and discussing the media's impact on stigma (*n* = 4).

Twenty-one studies (40%) involved someone with lived experience in the intervention development or delivery. This is in contrast to educational methods, which were used in almost all interventions (94%). No interventions reported using the protest as a method.

Of the 21 studies which involved someone with lived experience in intervention development or delivery, 86% were effective for all stigma-related outcomes compared to only 74% of studies which did not involve someone with lived experience. Of these 21, 15 incorporated emotional skills or emphasised recovery/strength of people with mental illness. In comparison, only five of the 31 studies which did not involve someone with lived experience incorporated emotional skills or emphasised recovery/strength.

#### Follow-up

**Fig. 3.**
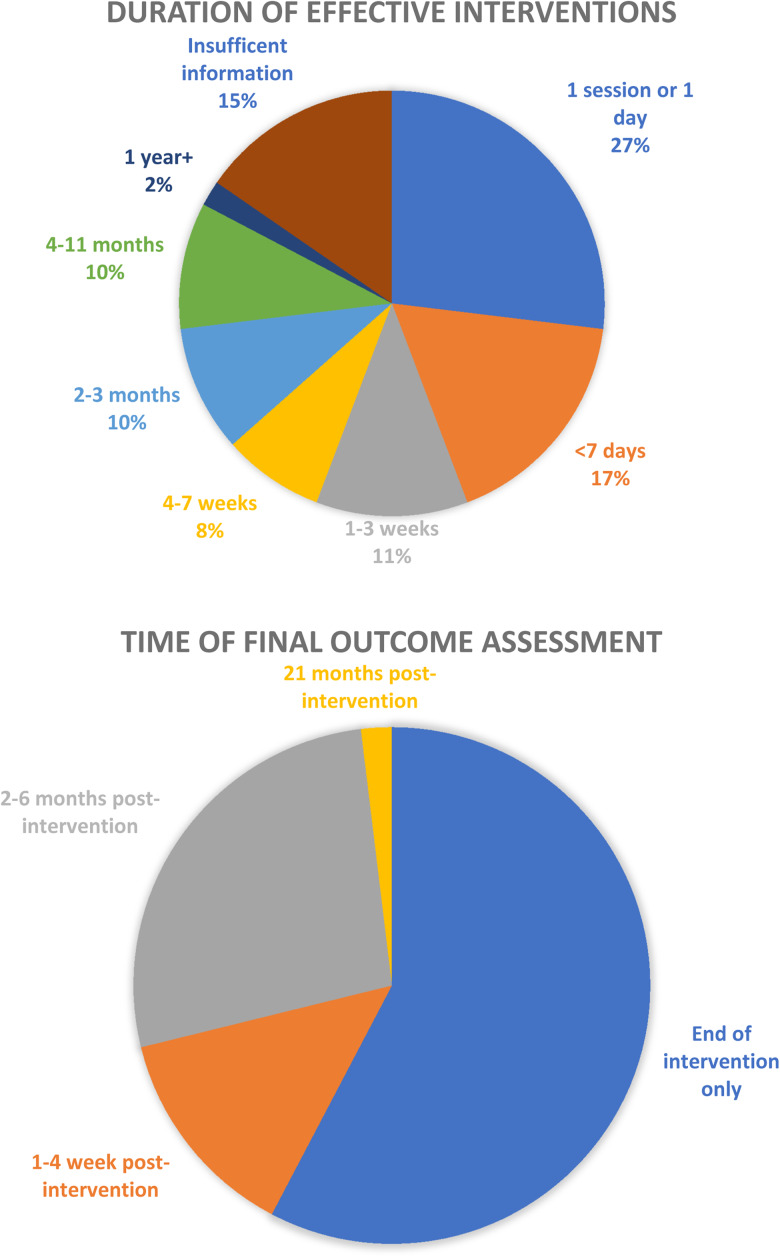
Duration of effective interventions and length of follow-up.

Approximately a quarter of interventions (*n* = 14) lasted for one session or 1 day only. Nine (16%) lasted for less than a week, six (10%) lasted between 1 and 3 weeks, and fifteen (29%) lasted 4 weeks or longer. The majority of studies (*n* = 30, 58%) had their final outcome assessment directly at the end of the intervention. However, of these 30, 14 interventions lasted 4 weeks or longer (see Fig. 3).

### Culture

Only 11 studies (21%) originated from ‘inside’ the setting (the source document for the intervention originated from the same setting it was used in) and were referenced (meaning the publication provided a cited resource for the intervention). Twelve studies (23%) were either ‘outside’ or new, had no reference and were not targeted/provided no targeting information. Thirty-five studies (67%) included a reference or evidence for their intervention. Thirty-two studies (62%) used interventions which originated ‘outside’ the country (such as source documents from the World Health Organisation) or did not provide information. Twenty-five studies (48%) were culturally adapted.

Only four of 52 studies (8%) took place in low-income countries; 21 (40%) took place in lower-middle-income countries and the remaining 27 (52%) took place in upper-middle-income countries. Given the geographic variety of results and as most regions were dominated by one country, analysis of components between World Bank regions was deemed an appropriate perspective on whether components vary between cultures.

Key intervention components did vary across regions (see Table 4). East Asia and Pacific had the highest number and percentage of: RCTs (*n* = 8, 62% of 13 studies) and interventions using emotional skills or emphasising recovery (*n* = 10, 76%). The region only utilised social contact in one study, however.

**Table 4.** Study components by geographical region

Region	*n*^a^	Study design				Target population				Average sample size at baseline	Intervention duration (where known)			Overall quality	
		Pre/post^b^	RCT	Non-randomised trial	Mixed methods	Health care workers	Students/ teachers	Family, caregivers, service users	The public		<1 week	1–4 weeks	>4 weeks	Poor/very poor	Moderate/high
East Asia and Pacific	13	3 (23%)	8 (62%)	2 (15%)	0 (0%)	3 (23%)	4 (31%)	6 (46%)	0 (0%)	170	5 (38%)	3 (23%)	3 (23%)	4 (31%)	9 (69%)
Sub-Saharan Africa	11	7 (64%)	1 (9%)	1 (9%)	2 (18%)	4 (36%)	6 (55%)	1 (9%)	0 (0%)	298	8 (73%)	0 (0%)	2 (18%)	3 (27%)	8 (73%)
South Asia	10	7 (70%)	1 (10%)	1 (10%)	1 (10%)	4 (40%)	3 (30%)	2 (20%)	2 (20%)	344	4 (40%)	1 (10%)	3 (30%)	1 (10%)	9 (90%)
Europe and Central Asia	7	2 (29%)	2 (29%)	3 (43%)	0 (0%)	1 (14%)	4 (57%)	2 (29%)	0 (0%)	117	3 (43%)	0 (0%)	4 (57%)	3 (43%)	4 (57%)
Latin America and the Caribbean	7	5 (71%)	1 (14%)	1 (14%)	0 (0%)	3 (43%)	2 (28%)	1 (14%)	1 (14%)	302	2 (29%)	1 (14%)	2 (29%)	2 (29%)	5 (71%)
Middle East and North Africa	4	2 (50%)	2 (50%)	0 (0%)	0 (0%)	1 (25%)	0 (0%)	3 (75%)	0 (0%)	128	2 (50%)	1 (25%)	1 (25%)	1 (25%)	3 (75%)

Region	*n*^a^	Intervention methods				Content detail				Lived exp^c^	Cultural adaptation	Reference/ evidence of source^d^	Final assessment: end only	Overall effectiveness for stigma-related outcomes	
		Education/ comms	Social contact	Creative/ tech	Therapeutic	Emotional	Recovery/ strength	Stigma	Myths					Sig. positive effect	Mixed results
East Asia and Pacific	13	11 (85%)	1 (8%)	5 (38%)	2 (15%)	5 (38%)	5 (38%)	7 (54%)	2 (15%)	7 (54%)	7 (54%)	8 (62%)	9 (69%)	10 (77%)	3 (23%)
Sub-Saharan Africa	11	11 (100%)	0 (0%)	7 (64%)	0 (0%)	1 (9%)	1 (9%)	5 (45%)	0 (0%)	1 (9%)	6 (55%)	9 (82%)	7 (64%)	8 (73%)	3 (27%)
South Asia	10	10 (100%)	3 (30%)	7 (70%)	0 (0%)	1 (10%)	2 (20%)	3 (30%)	3 (30%)	5 (50%)	6 (60%)	7 (70%)	3 (30%)	6 (60%)	4 (40%)
Europe and Central Asia	7	7 (100%)	3 (43%)	3 (43%)	0 (0%)	1 (14%)	2 (29%)	5 (71%)	3 (43%)	5 (71%)	1 (14%)	4 (57%)	3 (43%)	6 (86%)	1 (14%)
Latin America and the Caribbean	7	6 (86%)	0 (0%)	3 (43%)	0 (0%)	1 (14%)	2 (29%)	5 (71%)	1 (14%)	1 (14%)	3 (43%)	4 (57%)	5 (71%)	5 (71%)	2 (29%)
Middle East and North Africa	4	4 (100%)	0 (0%)	2 (50%)	0 (0%)	2 (50%)	1 (25%)	1 (25%)	0 (0%)	2 (50%)	2 (50%)	3 (75%)	2 (50%)	4 (100%)	0 (0%)

a Number of studies per region.

b Percentages are out of total studies in each region.

c Lived exp = involving someone with lived experience in intervention development/delivery.

d Reference/evidence of source = the origin of the intervention is referenced/evidence.

Sub-Saharan Africa had the lowest number and percentage of RCTs (*n* = 1, 9% of 11 studies), of interventions lasting longer than a week (*n* = 2, 18%), of interventions using emotional skills or emphasising recovery (*n* = 2, 18%), or involving someone with lived experience (*n* = 1, 9%). This region did have a good number of moderate and high-quality studies (*n* = 8, 73%), but no interventions featured social contact. This region also had the highest number of interventions whose source was referenced or evidenced (*n* = 9, 82%).

South Asia had the highest percentage of moderate or high-quality studies, at 90%. Europe and Central Asia had the most interventions lasting more than 1 month (*n* = 4, 57%). All regions were dominated by studies from one country: China (*n* = 7), India (*n* = 9), Nigeria (*n* = 5), Turkey (*n* = 5), Iran (n = 2) and Brazil (*n* = 5); combined they made up 63% (*n* = 33) of all effective studies.

### Ineffective studies

Only four (7%) of the 56 studies reported in Table 2 did not find any evidence of effectiveness and two were of poor or very poor methodological quality. These ineffective interventions did not use any social contact, technological or therapeutic methods. Two of the four provided no information on either delivery agents or their training/supervision and only one included explicit messaging on stigma or recovery. None involved someone with lived experience and only one was culturally adapted.

## Discussion

Overall, the vast majority of included studies were effective in reducing mental health stigma to some degree, at least in the short term, which is consistent with previous findings (Gronholm *et al*., 2017; Morgan *et al*., 2018).

There is some congruity in components between cultures, but generally, they vary widely and may reflect various cultural differences within the local setting. The generalisability of regional results to the wider World Bank regions is limited, as results were dominated by studies from one country in each region (China, India, Nigeria, Turkey, Iran and Brazil). Few studies originated from the local setting and were referenced and less than half met the criteria to be considered culturally adapted. This assumption of transferability between settings by some studies disregards the importance of cultural relevance (Drake *et al*., 2014; Rathod and Kingdon, 2014) and evidence that local concepts of stigma are complex (Weiss *et al*., 2001).

Despite evidence of the effectiveness of social contact (Corrigan and Scott, 2012), no region overtly described using social contact in more than half of studies and almost no studies defined any of their delivery agents as someone with lived experience. Nonetheless, it is possible that social contact was more frequently used and included in other intervention components, such as through lectures and presentations. However, if not explicitly mentioned, it was unable to be reported here.

The results of this review also indicate that more complex interventions (i.e. interventions including more unique individual methodological sub-components and elements) are not necessarily more effective. Almost all studies used self-report measures and had limited length of follow-up, which reflects the complexity of measuring real-life discrimination experience, hence use of, for example, intended behaviour as proxies.

The results of this systematic review reiterate some main findings from other recent stigma-related reviews (Thornicroft *et al*., 2016; Heim *et al*., 2018; Mehta *et al*., 2018; Morgan *et al*., 2018; Heim *et al*., 2019). However, these reviews also found minimal research in LMICs, limited cultural adaptation, short intervention duration, poor study quality and mostly only short-term follow-up. Yet in comparison, this review found a higher number of effective studies with more than a 4-week follow-up, the majority of which were of moderate or high quality and more studies which originated from inside the local setting. The majority of papers included here have been published since 2014. This indicates that the overall quantity and quality of stigma reduction studies has increased over the past several years.

### Strengths and limitations

This is the first systematic review providing an in-depth analysis of core components of mental health stigma reduction interventions in LMICs.

The relatively large number of included studies provides a thorough analysis of intervention components and the use of an explicit evidence-based framework allows for more definitive conclusions on the makeup and distribution of various intervention components in LMICs and within specific regions. This review also includes an emphasis on interrogating culture and adaptation, which is valuable given the importance of cultural understanding. Although there were a large number of non-randomised trials and pre/post studies, this review included a higher percentage of RCTs than previous reviews in LMICs, improving the overall quality of results.

One limitation was only including studies with full-texts in English or Spanish, the languages spoken of the author. The components reported here were selected based on criteria established through the framework synthesis, but detailed analysis of additional study characteristics would be useful and interesting. Additionally, studies without full-texts available were excluded and only two authors replied to full-text requests.

Core components were extracted and included only if they were explicitly reported in the publication and although authors were contacted whenever possible for further detail most did not reply. Additionally, more than half the studies were pre/post studies without a control group, increasing the possibility of bias and a quarter of studies were of poor or very poor quality. Regarding stigma reduction measures, although evidence surrounding knowledge gain as a proxy for stigma is mixed (Stuart, 2016), interventions solely measuring knowledge gain were included (*n* = 1) as per a reflection of the existing stigma literature. Further research is needed to understand what aspects of knowledge-based interventions impact stigma and how. The generalisability of the region-specific analysis was reduced by the fact that each region's studies were dominated by a single country. Finally, the small number of ineffective studies suggests that there may be publication bias in this area of research.

### Implications and recommendations

The vast amount of extracted data for this review, organised into 106 detailed components, sub-components and elements, should provide other researchers with a useful starting point for designing and analysing mental health stigma reduction interventions.

Given that the majority of stigma research focuses on HICs, understanding what has worked in varied low-resource settings would be essential when developing effective stigma reduction interventions in LMICs, for example when using creative methods such as theatre, dance and web-based interventions.

More research needs to be conducted in a wider variety of countries, and interventions need to be developed using local expertise and be culturally adapted. Due to its proven effectiveness (Gronholm *et al*., 2017), social contact should be actively incorporated into stigma reduction interventions.

## Conclusion

This systematic review found many and varied stigma reduction programmes with effective intervention components in LMICs. Most included studies described interventions based on educational methods, along with themes of medical knowledge surrounding mental health, teaching emotional/communication skills, myth-busting, emphasising recovery and discussing the media's impact on stigma. Yet there are minimal descriptions of social contact, despite the fact that it has been shown to be the most impactful single component of stigma reduction work (Thornicroft *et al*., 2016). This may be due to lack of knowledge about the state of evidence around contact interventions, lack of influence and opportunity for groups of people affected to be included, or stigma itself.

Although to date only a minority of studies were developed, evidenced or culturally adapted in the local setting, the overall quantity and quality of studies has increased over the past several years. If the best evidence was available to groups working to combat stigma globally, it is likely that the important benefits of these efforts to promote inclusion and reduce stigma and discrimination would be amplified, and more people in need would seek and get access to mental health care.

## Supplementary material

For supplementary material accompanying this paper visit http://dx.doi.org/10.1017/S2045796020000797. click here to view supplementary material

## Availability of Data and Materials

For access to all data supporting this study, please email Jennifer Clay: jeclay3@gmail.com.
