# Supplementary material for: Core components of mental health stigma reduction interventions in low- and middle-income countries: a systematic review
Source: Epidemiol Psychiatr Sci. 2020 Sep 4;29:e164. doi: 10.1017/S2045796020000797 (PMC7503169; doi:10.1017/S2045796020000797)
Supplement: Supplementary file 1 [file S2045796020000797sup001.docx]

## Supplementary material

## 1.1: Priority inclusion order tool for study selection

- - 1. Studies must relate to any type of *mental health stigma or discrimination* (including social stigma, self-stigma, anticipated, perceived, or experienced stigma or discrimination), or related to *negative attitudes, beliefs or behaviours* on mental health
  - *Exclude***:** studies solely focused on the stigma of:
    - - Neurological conditions
      - Substance misuse
      - Other health conditions (HIV, leprosy)
      - Other social characteristics (gender, sexual orientation)

1. Must be an *intervention*—all experimental designs included
   - *Exclude***:** observational study designs
   - *Stigma reduction of any definition* must be an outcome
2. Must take place in a *LMIC*
   - *Exclude***:** HIC
3. Must have a *stigma-related outcome measure*
   - E.g. Bogardus Social Distance Scale

## 1.2: MEDLINE search terms

| A: Stigma | B: Mental health | C: Intervention | D: LMIC | E: Limits |
| --- | --- | --- | --- | --- |
| Subject headings: | exp Mental Disorders/ | Health education/ | Afghanistan or Albania or Algeria or Samoa or Angola or Antigua or Barbuda or Argentina or Armenia or Azerbaijan or Bangladesh or Belarus or Belize or Benin or Bhutan or Bolivia or Bosnia or Herzegovina or Botswana or Brazil or Bulgaria or “Burkina Faso” or Burundi or Cambodia or Cameroon or “Cabo Verde” or “Central African Republic” or Chad or Chile or China or Colombia or Comoros or Congo or “Costa Rica” or Cote or Ivoire or Ivory or Cuba or Djibouti or Dominica or Dominican or Ecuador or Egypt or Salvador or Eritrea or Ethiopia or Fiji or Gabon or Gambia or Georgia or Ghana or Grenada or Guatemala or Guinea or Guinea-Bissau or Guyana or Haiti or Honduras or India or Indonesia or Iran or Iraq or Jamaica or Jordan or Kazakhstan or Kenya or Kiribati or Korea or Kosovo or Kyrgyz or Lao or Laos or Latvia or Lebanon or Lesotho or Liberia or Libya or Lithuania or Macedonia or Madagascar or Malawi or Malaysia or Maldives or Mali or Marshall or Mauritania or Mauritius or Mexico or Micronesia or Moldova or Mongolia or Montenegro or Morocco or Mozambique or Myanmar or Namibia or Nepal or Nicaragua or Niger or Nigeria or Pakistan or Palau or Panama or “Papua New Guinea” or Paraguay or Peru or Philippines or Romania or Russia or Russian or Rwanda or Samoa or “Sao Tome” or Senegal or Serbia or Seychelles or “Sierra Leone” or “Solomon Island” or Somalia or “South Africa” or “Sri Lanka” or “St Lucia” or "Saint Lucia" or “St Vincent” or “Saint Vincent” or Grenadines or Sudan or Suriname or Swaziland or Syrian or Syria or Tajikistan or Tanzania or Thailand or Timor-Leste or Togo or Tonga or Tunisia or Turkey or Turkmenistan or Tuvalu or Uganda or Ukraine or Uruguay or Uzbekistan or Vanuatu or Venezuela or Vietnam or “West Bank” or Gaza or Palestine or Yemen or Zambia or Zimbabwe | humans |
| "Discrimination (Psychology)/" | Mental Health/ | Health literacy/ |  | English or Spanish |
| prejudice/ | Mentally Ill Persons/ | Community Mental Health Services/ |  |  |
| "rejection (psychology)/" | suicide/ | Evaluation studies/ |  |  |
| social discrimination/ | Keywords (.mp): | Program Evaluation/ |  |  |
| social distance/ | mental disorder* | Follow-up Studies/ |  |  |
| social marginalization/ | mental health | Health Knowledge, Attitudes, Practice/ |  |  |
| social isolation/ | mental hygiene | Intervention Studies/ |  |  |
| social stigma/ | suicide | Randomized Controlled Trials as Topic/ |  |  |
| stereotyping/ | mental* ill* | Keywords (.mp): |  |  |
| Stereotyped Behavior/ | psychotic | clinical trial |  |  |
| Attitude/ | psychosis | program* adj5 effectiv* |  |  |
| Attitude of Health Personnel/ |  | stigma* adj5 reduc* |  |  |
| Keywords (.mp): |  | discrim* adj5 reduc* |  |  |
| discriminat* |  | campaign* |  |  |
| prejudice |  | initiative* |  |  |
| social discriminat* |  | awareness |  |  |
| social distanc* |  | health literacy |  |  |
| social marginali* |  | intervent* |  |  |
| social stigma* |  | training |  |  |
| stereotyp* |  | (improve* OR change*) adj5 (knowledge* OR attitude* or behavio*) |  |  |
| stereotyp* behavio* |  | anti-stigma* |  |  |
| embarass* |  | workshop* |  |  |
| stigma* |  | community adj5 "mental health" |  |  |
| social acceptance |  | antistigma |  |  |
| social perception |  | contact-based |  |  |
| social isolation |  | change adj5 stigma |  |  |
|  |  | pre-post |  |  |
|  |  | evaluat* | OR “developing countr*” or resource-limited or resource-constrained or “low- and middle-income” or “low and middle-income” or LMIC or “third world” or “low-income” or “middle-income”.mp. |  |
|  |  |  |  |  |
|  |  |  |  |  |
|  |  |  |  |  |
|  |  |  | OR Afghanistan/ OR Albania/ OR Algeria/ OR American Samoa/ OR Angola/ OR Argentina/ OR Armenia/ OR Azerbaijan/ OR Bangladesh/ OR Belarus/ OR Belize/ OR Benin/ OR Bhutan/ OR Bolivia/ OR "Bosnia and Herzegovina"/ OR Botswana/ OR Brazil/ OR Bulgaria/ OR "Burkina Faso"/ OR Burundi/ OR Cape Verde/ OR Cambodia/ OR Cameroon/ OR Central African Republic/ OR Chad/ OR China/ OR Colombia/ OR Comoros/ OR Congo/ OR Costa Rica/ OR Cote d'Ivoire/ OR Cuba/ OR Djibouti/ OR Dominica/ OR Dominican Republic/ OR Ecuador/ OR Egypt/ OR El Salvador/ OR Equatorial Guinea/ OR Eritrea/ OR Ethiopia/ OR Fiji/ OR Gabon/ OR Gambia/ OR Georgia/ OR Ghana/ OR Grenada/ OR Guatemala/ OR Guinea/ OR Guinea-Bissau/ OR Guyana/ OR Haiti/ OR Honduras/ OR India/ OR Indonesia/ OR Iran/ OR Iraq/ OR Jamaica/ OR Jordan/ OR Kazakhstan/ OR Kenya/ OR Kiribati/ OR "Democratic People's Republic of Korea"/ OR Kosovo/ OR Kyrgyzstan/ OR Laos/ OR Lebanon/ OR Lesotho/ OR Liberia/ OR Libya/ OR "Macedonia (Republic)"/ OR Madagascar/ OR Malawi/ OR Malaysia/ OR Maldives/ OR Mali/ OR Marshall Islands.mp. OR Mauritania/ OR Mauritius/ OR Mexico/ OR Micronesia/ OR Moldova/ OR Mongolia/ OR Montenegro/ OR Morocco/ OR Mozambique/ OR Myanmar/ OR Namibia/ OR Nepal/ OR Nicaragua/ OR Niger/ OR Nigeria/ OR Pakistan/ OR Palau/ OR Panama/ OR Papua New Guinea/ OR Paraguay/ OR Peru/ OR Philippines/ OR Romania/ OR Russia/ OR Rwanda/ OR Samoa/ OR (Sao Tome and Principe).mp OR Senegal/ OR Serbia/ OR Sierra Leone/ OR Melanesia/ OR Somalia/ OR South Africa/ OR South Sudan/ OR Sri Lanka/ OR Saint Lucia/ OR "Saint Vincent and the Grenadines"/ OR Sudan/ OR Suriname/ OR Swaziland/ OR Syria/ OR Tajikistan/ OR Tanzania/ OR Thailand/ OR Timor-Leste/ OR Togo/ OR Tonga/ OR Tunisia/ OR Turkey/ OR Turkmenistan/ OR Tuvalu.mp OR Uganda/ OR Ukraine/ OR Uzbekistan/ OR Vanuatu/ OR Venezuela/ OR Vietnam/ OR Yemen/ OR Zambia/ OR Zimbabwe/ |  |
|  |  |  |  |  |
|  |  |  |  |  |
|  |  |  |  |  |
|  |  |  |  |  |
|  |  |  |  |  |
|  |  |  |  |  |
|  |  |  |  |  |
|  |  |  |  |  |
|  |  |  |  |  |
|  |  |  |  |  |
|  |  |  |  |  |
|  |  |  |  |  |
|  |  |  |  |  |
|  |  |  |  |  |
|  |  |  |  |  |
|  |  |  |  |  |
|  |  |  |  |  |
|  |  |  |  |  |
|  |  |  |  |  |
|  |  |  |  |  |
|  |  |  |  |  |
|  |  |  |  |  |
|  |  |  |  |  |
|  |  |  |  |  |
|  |  |  |  |  |
|  |  |  |  |  |
|  |  |  |  |  |
|  |  |  |  |  |
|  |  |  |  |  |

## 1.3 Data extraction worksheet fields

| General Information | | |  |  |  |
| --- | --- | --- | --- | --- | --- |
| Study ID | | |  |  |  |
| Date of data extraction | | |  |  |  |
| Title | | |  |  |  |
| Authors | | |  |  |  |
| Year of publication | | |  |  |  |
| General study characteristics | | |  |  |  |
| Study design | | |  |  |  |
| Country | | |  |  |  |
| City or town; setting | | |  |  |  |
| Primary aim | | |  |  |  |
| Secondary aims | | |  |  |  |
| Target population | | |  |  |  |
| Target type of stigma | | |  |  |  |
| Study methods | | |  |  |  |
| When was it delivered? | | |  |  |  |
| Mean age (all participants) | | |  |  |  |
| % female (participants) | | |  |  |  |
| Education (participants) | | |  |  |  |
| Sample size (total, at start) | | |  |  |  |
| Sample size (intervention) | | |  |  |  |
| Sample size (control) | | |  |  |  |
| Other relevant socio-demographics (participants) | | |  |  |  |
| Additional cultural or contextual information | | |  |  |  |
| Inclusion criteria | | |  |  |  |
| Exclusion criteria | | |  |  |  |
| Method of recruitment | | |  |  |  |
| Follow up time points (apart from baseline) | | |  |  |  |
| Intervention characteristics | | |  |  |  |
| Intervention rationale, theory or origin | | |  |  |  |
| Main intervention methods/components (presentations, workshops, roleplay, Q&A, discussion, etc) | | |  |  |  |
| Type (contact vs education vs protest) | | |  |  |  |
| Group size | | |  |  |  |
| Duration per session | | |  |  |  |
| frequency/duration overall | | |  |  |  |
| Dissemination mediums (e.g. face-to-face, leaflets, video) | | |  |  |  |
| Delivery agent(s)- qualifications | | |  |  |  |
| Delivery agent(s)- training/supervision for intervention | | |  |  |  |
| Service user/lived experience involvement? | | |  |  |  |
| Content detail: social content, myth busting, education, emphasis on recovery, etc? | | |  |  |  |
| Cultural adaptation? Are local beliefs taken into account? | | |  |  |  |
| Economic info (inc. cost or benefit to participants) | | |  |  |  |
| Resource requirements (e.g. staff numbers, equipment) | | |  |  |  |
| Use of technology? (beyond basic laptop functions) | | |  |  |  |
| Important discussion points | | |  |  |  |
| Other intervention notes (JC) | | |  |  |  |
| Results | | |  |  |  |
| Stigma outcome 1: scale/measure | | |  |  |  |
| Outcome 1: definition/detail | | |  |  |  |
| Outcome 1: measure validation? | | |  |  |  |
| Outcome 1: baseline mean- time pt 1 (SD) | | |  |  |  |
| Outcome 1: post-intervention mean- time pt 2 (SD) | | |  |  |  |
| Outcome 1: follow-up mean- time pt 3 (SD) | | |  |  |  |
| Main results found for stigma outcome 1 | | |  |  |  |
| Stigma outcome 2: scale/measure | | |  |  |  |
| Outcome 2: definition | | |  |  |  |
| Outcome 2: measure validation? | | |  |  |  |
| Outcome 2: baseline mean- time pt 1 (SD) | | |  |  |  |
| Outcome 2: post-intervention mean- time pt 2 (SD) | | |  |  |  |
| Outcome 2: follow-up mean- time pt 3 (SD) | | |  |  |  |
| Main results found for stigma outcome 2 | | |  |  |  |
| Stigma outcome 3: scale/measure | | |  |  |  |
| Outcome 3: definition | | |  |  |  |
| Outcome 3: measure validation? | | |  |  |  |
| Main results found for stigma outcome 3 | | |  |  |  |
| % (#) complete outcome data (all time points) | | |  |  |  |
| Overall effectiveness: for stigma-related outcomes | | |  |  |  |
| Evidence of effectiveness (any size)? | | |  |  |  |
| Other notes on results (JC, or "" if from text) | | |  |  |  |
| Other | | |  |  |  |
| Other comments (limitations, funding, etc) | | |  |  |  |
| Quality assessment summary | | |  |  |  |
| % (#) complete outcome data (all time points) | | |  |  |  |
| % of criteria met | | |  |  |  |
| Overall quality | | |  |  |  |
| Outside of quality: Relevance and/or level of argument for study, if of note | | |  |  |  |
| Comments | | |  |  |  |
| Quality Assessment (MMAT) | | | | | |
| *Qualitative* | *Quantitative RCT* | *Quantitative non-randomized* | | *Quantitative descriptive* | *Mixed methods* |
| 1.1. Is the qualitative approach appropriate to answer the research question? | 2.1. Is randomization appropriately performed? | 3.1. Are the participants representative of the target population? | | 4.1. Is the sampling strategy relevant to address the research question? | 5.1. Is there an adequate rationale for using a mixed methods design to address the research question? |
| 1.2. Are the qualitative data collection methods adequate to address the research question? | 2.2. Are the groups comparable at baseline? | 3.2. Are measurements appropriate regarding both the outcome and intervention (or exposure)? | | 4.2. Is the sample representative of the target population? | 5.2. Are the different components of the study effectively integrated to answer the research question? |
| 1.3. Are the findings adequately derived from the data? | 2.3. Are there complete outcome data? (No=<80% for now) | 3.3. Are there complete outcome data? (No=<80% for now) | | 4.3. Are the measurements appropriate? | 5.3. Are the outputs of the integration of qualitative and quantitative components adequately interpreted? |
| 1.4. Is the interpretation of results sufficiently substantiated by data? | 2.4. Are outcome assessors blinded to the intervention provided? | 3.4. Are the confounders accounted for in the design and analysis? | | 4.4. Is the risk of nonresponse bias low? | 5.4. Are divergences and inconsistencies between quantitative and qualitative results adequately addressed? |
| 1.5. Is there coherence between qualitative data sources, collection, analysis and interpretation? | 2.5 Did the participants adhere to the assigned intervention? | 3.5. During the study period, is the intervention administered (or exposure occurred) as intended? | | 4.5. Is the statistical analysis appropriate to answer the research question? | 5.5. Do the different components of the study adhere to the quality criteria of each tradition of the methods involved? |

## 1.4 Related stigma reviews

1. Alonso M, Guillén AI, Muñoz M. Interventions to Reduce Internalized Stigma in individuals with Mental Illness: A Systematic Review. The Spanish Journal of Psychology. 2019;22:E27.
2. Ando S, Clement S, Barley EA, Thornicroft G. The simulation of hallucinations to reduce the stigma of schizophrenia: a systematic review. Schizophr Res. 2011;133(1-3):8-16.
3. Büchter RB, Messer M. Interventions for reducing self-stigma in people with mental illnesses: a systematic review of randomized controlled trials. German medical science : GMS e-journal. 2017;15:Doc07-Doc.
4. Candidate number: 110525. Systematic Review of Stigma Reduction Interventions in Low- and Middle-Income Countries. Unpublished dissertation- LSHTM Library. 2018.
5. Clement S, Lassman F, Barley E, Evans-Lacko S, Williams P, Yamaguchi S, et al. Mass media interventions for reducing mental health-related stigma. Cochrane Database Syst Rev. 2013(7):CD009453.
6. Corrigan PM, Scott B. Challenging the Public Stigma of Mental Illness: A Meta-Analysis of Outcome Studies. Psychiatric Services. 2012.
7. Geffner N, Zalazar V, Mascayano F, Agrest M. Review of Anti-Stigma and Pro-Recovery Programs. ACTA PSIQUIATRICA Y PSICOLOGICA DE AMERICA LATINA. 2017;63(3):189-202.
8. Griffiths KM, Carron‐Arthur B, Parsons A, Reid R. Effectiveness of programs for reducing the stigma associated with mental disorders. A meta‐analysis of randomized controlled trials. World psychiatry. 2014;13(2):161-75.
9. Gronholm PC, Henderson C, Deb T, Thornicroft G. Interventions to reduce discrimination and stigma: the state of the art. Social Psychiatry and Psychiatric Epidemiology. 2017;52(3):249-58.
10. Hadlaczky G, Hökby S, Mkrtchian A, Carli V, Wasserman D. Mental Health First Aid is an effective public health intervention for improving knowledge, attitudes, and behaviour: A meta-analysis. International Review of Psychiatry. 2014;26(4):467-75.
11. Hanisch SE, Twomey CD, Szeto ACH, Birner UW, Nowak D, Sabariego C. The effectiveness of interventions targeting the stigma of mental illness at the workplace: a systematic review. BMC Psychiatry. 2016;16(1):1.
12. Heim E, Henderson C, Kohrt BA, Koschorke M, Milenova M, Thornicroft G. Reducing mental health-related stigma among medical and nursing students in low- and middle-income countries: a systematic review. Epidemiology and Psychiatric Sciences. 2019:1-9.
13. Heim E, Kohrt BA, Koschorke M, Milenova M, Thronicroft G. Reducing mental health-related stigma in primary health care settings in low- and middle-income countries: a systematic review. Epidemiology and Psychiatric Sciences. 2018:1-10.
14. Kemp CG, Jarrett BA, Kwon C-S, Song L, Jetté N, Sapag JC, et al. Implementation science and stigma reduction interventions in low- and middle-income countries: a systematic review. BMC Medicine. 2019;17(1):6.
15. Liu G, Jack H, Piette A, Mangezi W, Machando D, Rwafa C, et al. Mental health training for health workers in Africa: a systematic review. The Lancet Psychiatry. 2016;3(1):65-76.
16. Mansouri N, Gharaee B, Shariat SV, Bolhari J, Yousefi Nooraie R, Rahimi-Movaghar A, et al. The change in attitude and knowledge of health care personnel and general population following trainings provided during integration of mental health in Primary Health Care in Iran: a systematic review. International Journal of Mental Health Systems. 2009;3(1):15.
17. Mehta N, Clement S, Marcus E, Stona AC, Bezborodovs N, Evans-Lacko S, et al. Evidence for effective interventions to reduce mental health-related stigma and discrimination in the medium and long term: Systematic review. British Journal of Psychiatry. 2018;207(05):377-84.
18. Mellor C. School-based interventions targeting stigma of mental illness: systematic review. The Psychiatric Bulletin. 2018;38(4):164-71.
19. Morgan AJ, Reavley NJ, Ross A, Too LS, Jorm AF. Interventions to reduce stigma towards people with severe mental illness: Systematic review and meta-analysis. J Psychiatr Res. 2018;103:120-33.
20. Navarro Esquerda A. Intervenciones para disminuir el autoestigma en personas con TMG. Revisión bibliográfica. 2018.
21. Rao D, Elshafei A, Nguyen M, Hatzenbuehler ML, Frey S, Go VF. A systematic review of multi-level stigma interventions: state of the science and future directions. BMC Medicine. 2019;17(1):41.
22. Seroalo KB, Du Plessis E, Koen MP, Koen V. A critical synthesis of interventions to reduce stigma attached to mental illness. Health SA Gesondheid. 2014;19(1).
23. Thornicroft G, Mehta N, Clement S, Evans-Lacko S, Doherty M, Rose D, et al. Evidence for effective interventions to reduce mental-health-related stigma and discrimination. The Lancet. 2016;387(10023):1123-32.
24. Xu Z, Huang F, Kösters M, Rüsch N. Challenging mental health related stigma in China: Systematic review and meta-analysis. II. Interventions among people with mental illness. Psychiatry Research. 2017;255:457-64.
25. Xu Z, Rüsch N, Huang F, Kösters M. Challenging mental health related stigma in China: Systematic review and meta-analysis. I. Interventions among the general public. Psychiatry Research. 2017;255:449-56.
